# SNP-revealed genetic diversity in wild emmer wheat correlates with ecological factors

**DOI:** 10.1186/1471-2148-13-169

**Published:** 2013-08-13

**Authors:** Jing Ren, Liang Chen, Daokun Sun, Frank M You, Jirui Wang, Yunliang Peng, Eviatar Nevo, Avigdor Beiles, Dongfa Sun, Ming-Cheng Luo, Junhua Peng

**Affiliations:** 1 Key Laboratory of Plant Germplasm Enhancement and Specialty Agriculture, Chinese Academy of Sciences, Wuhan, Hubei 430074, China; 2 Graduate University of Chinese Academy of Sciences, Beijing 100049, China; 3 Department of Plant Sciences, University of California, Davis, CA 95616, USA; 4 Cereal Research Centre, Agriculture and Agri-Food Canada, Winnipeg, MB R3T 2M9, Canada; 5 Institute of Plant Protection, Sichuan Academy of Agricultural Sciences, Chengdu, Sichuan 610066, China; 6 Institute of Evolution, University of Haifa, Mount Carmel, Haifa 31905, Israel; 7 College of Plant Science & Technology, Huazhong Agricultural University, Wuhan Hubei 430071, China; 8 Department of Soil and Crop Sciences, Colorado State University, Fort Collins, CO 80523, USA

**Keywords:** *Triticum dicoccoides*, SNP marker, Adaptive genetic diversity, Population structure, Natural selection

## Abstract

**Background:**

Patterns of genetic diversity between and within natural plant populations and their driving forces are of great interest in evolutionary biology. However, few studies have been performed on the genetic structure and population divergence in wild emmer wheat using a large number of EST-related single nucleotide polymorphism (SNP) markers.

**Results:**

In the present study, twenty-five natural wild emmer wheat populations representing a wide range of ecological conditions in Israel and Turkey were used. Genetic diversity and genetic structure were investigated using over 1,000 SNP markers. A moderate level of genetic diversity was detected due to the biallelic property of SNP markers. Clustering based on Bayesian model showed that grouping pattern is related to the geographical distribution of the wild emmer wheat. However, genetic differentiation between populations was not necessarily dependent on the geographical distances. A total of 33 outlier loci under positive selection were identified using a *F*_*ST*_-outlier method. Significant correlations between loci and ecogeographical factors were observed.

**Conclusions:**

Natural selection appears to play a major role in generating adaptive structures in wild emmer wheat. SNP markers are appropriate for detecting selectively-channeled adaptive genetic diversity in natural populations of wild emmer wheat*.* This adaptive genetic diversity is significantly associated with ecological factors.

## Background

Patterns of genetic diversity between and within natural plant populations and their driving forces are of great interest in evolutionary biology, as well as in studies of ecological and population genetics (Nevo list of wild cereals at http://evolution.haifa.ac.il) [1,2]. The analyses of genetic diversity and structure are helpful for management, research and utilization of plant germplasm. It is also critical for studies of crop evolution and genetic improvement to identify and correctly interpret the associations between functional variation and molecular genetic diversity [2,3]. Wild emmer wheat, *Triticum dicoccoides,* has been found in a wide range of environments, and shows high genetic and phenotypic diversity [3]. The analysis of the genetic structure and population divergence of such high diversity is important for breeding purposes, especially to identify genes or genomic regions involved in environmental adaptation. Furthermore, wheat serves as a good model of polyploidy, one of the most common forms of plant evolution [4,5]. Hence, it is cardinal to study adaptive genetic diversity in wild emmer, the progenitor of modern tetraploid and hexaploid cultivated wheats [1,2,6,7].

Wild emmer wheat, *T. dicoccoides* (2n = 4x = 28, genome AABB), is a tetraploid predominantly self-pollinated plant. It originated from a spontaneous hybridization of wild diploid einkorn wheat, *T. urartu* (2n = 2x = 14, genome AA), with a close relative of the goat grass *Aegilops speltoides* (2n = 2x = 14, genome SS, where S is closely related to B) [8,9]. Wild emmer wheat presumably originated in and adaptively diversified from north-eastern Israel and the Golan into the Near East Fertile Crescent, across a variety of ecological conditions [10]. The wide range of ecological conditions, such as temperature [1,11], soil [1,12], water availability [1,10], light intensity [1,11], humidity [1,13-16], etc., may exert diverse selection pressures, thus determine the evolutionary course while shaping its genetic structure. Wild emmer wheat has adapted to a broad range of environments and is rich in genetic resources that include drought and salt tolerances [10,17], herbicide tolerances [1,18], Zn and Fe contents [19,20], biotic (viral, bacterial, and fungal) tolerances [1,21], high-quantity and high-quality storage proteins [20], and many others. They represent one of the best hopes for crop improvement. Hence, genetic studies of wild emmer wheat are of paramount importance for wheat improvement.

In previous studies, genetic diversity of wild emmer wheat populations has been evaluated using various methods such as morphological traits [1,17], allozyme analysis [1,3,13], and many molecular markers (SSRs, RAPDs, and SRAPs) [14,15,22]. Association between markers and ecogeographical factors were also discussed [13-15,22]. However, genetic structure and population divergence revealed by EST-related SNP markers have not been reported in wild emmer wheat populations. EST-related markers discovered directly from the EST sequences or from genomic sequences amplified using PCR primers designed from ESTs, are useful resources for assaying functional genetic variation [23]. Variation in functional regions, expressed or regulatory sequence, might reflect the past influences of natural selection. Besides, because this type of SNPs can be linked to functional genes, it is important to determine which markers have been likely associated with selection, especially to identify genes or genomic regions involved in environmental adaptation. Hence, SNP markers seem the best to meet needs of marker-assisted management of genetic resources, and of diversity studies and marker-assisted selection in breeding programs. At present, the majority of studies using these EST-related SNP markers have focused on model organisms [24,25] with fewer applications to non-model taxa [26]. Only a limited number of SNPs have been reported in wheat [27-30]. Large-scale SNP discovery in wheat is limited by both the polyploidy nature of the organism and the high sequence similarity found among the three homoeologous wheat genomes [29,31].

In the present study, a large number of EST-related SNP markers were used to investigate genetic diversity and genetic structure of a natural collection of 200 accessions belonging to 25 wild emmer wheat populations. This germplasm was collected by E. Nevo from various locations in Israel and Turkey, which covers a wide range of ecological conditions such as soil, temperature, and water availability. Noteworthy, a *F*_*ST*_-outlier method was used to identify loci that may be under positive selection and therefore might be linked to genome regions conferring the phenotypic variation present in analyzed germplasm for breeding programs.

## Methods

### Plant materials and ecological background of wild emmer wheat

**Figure 1:**
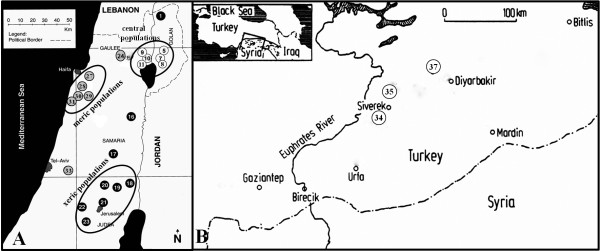
**Geographic distribution of 25 wild emmer wheat populations in Israel and Turkey. (A)** 22 populations in Israel, and **(B)** 3 populations in Turkey. Details of the numbered populations are listed in Table 1.

The center of distribution and diversity of emmer wheat was found in the catchment area of the upper Jordan Valley in Israel and its vicinity [13]. A total of 200 wild emmer wheat accessions representing 25 populations collected from Israel and Turkey (five to ten accessions per population) were used in this study. The plant materials originated from a wide range of ecological conditions of soil, temperature and water availability, representing the natural distribution of wild emmer wheat. Geographical locations of all the investigated populations are shown in Figure 1. The populations used in this study, along with their geographic origin and climatic conditions, are presented in Table 1. The Israeli climatic data was obtained from publications of the Meteorological Service of Israel [13]. Detailed information about each population and their collection sites have been described in the literatures (Nevo list of wild cereals at http://evolution.haifa.ac.il) [13-15].

**Table 1:** Ecogeographical variables for 25 wild emmer wheat populations in Israel and Turkey (see Nevo and Beiles 1989)

**Pop code**	**Place of origin**	**Ln**	**Lt**	**Al**	**Tm**	**Ta**	**Tj**	**Td**	**Tdd**	**Rn**	**Rd**	**Hu14**	**Huan**	**Dw**	**Trd**	**Ev**	**So**	**Rv**	**Rr**
01	Mt. Hermon	35.73	33.30	1300	11	21	3	18	6	1400	66	48	60	60	0	150	1	30	20
05	Qazrin	35.67	32.99	350	18	26	10	16	12	530	50	43	58	58	60	155	5	39	26
07	Yehudiyya	35.70	32.93	200	19	27	11	16	12	550	47	42	58	58	100	160	5	39	26
08	Gamla	35.74	32.88	200	19	26	9	17	12	470	50	43	58	58	60	155	5	39	26
09	Rosh-Pinna	35.52	32.95	700	18	25	9	16	10	679	50	48	58	50	35	150	1	35	22
10	Ammiad	35.50	32.90	270	19	26	10	16	10	700	48	48	58	50	50	150	1	38	25
11	Tabigha	35.53	32.90	0	24	32	15	17	10	436	45	45	57	58	120	160	5	39	25
16	Mt. Gilboa	35.42	32.50	150	21	28	12	16	12	400	43	43	58	40	160	165	1	34	24
17	Mt. Gerizim	35.28	32.20	800	17	23	8	15	9	700	45	45	60	42	0	155	1	38	25
18	Gitit	35.40	32.10	300	21	29	13	16	12	360	39	39	55	25	100	170	1	38	24
19	Kokhav-Hashahar	35.34	31.95	600	20	28	12	16	12	400	45	45	59	30	25	165	1	38	22
20	Taiyiba	35.35	31.92	450	19	26	10	16	12	400	40	44	58	30	25	165	1	38	22
21	Sanhedriyya	35.22	31.80	800	17	24	9	15	9	548	44	51	62	44	0	155	1	30	21
22	Bet-Meir	35.03	31.80	500	19	26	11	15	9	582	44	47	60	61	100	160	1	33	25
23	J’aba	35.08	31.67	660	17	25	9	15	9	500	49	49	62	57	30	155	1	35	21
24	Amirim	35.45	32.93	600	15	24	8	16	8	850	48	48	60	53	13	153	1	35	23
27	Nesher	35.05	32.75	200	19	26	12	14	8	680	55	57	68	82	5	140	1	27	19
28	Beit-Oren	35.03	32.73	400	17	24	11	13	8	700	59	59	69	80	0	143	1	25	19
29	Daliyya	35.06	32.59	200	19	26	12	14	11	670	57	57	67	78	100	160	2	25	20
30	Bat-Shelomo	35.02	32.60	75	20	26	13	13	10	650	58	58	68	77	30	150	2	24	20
31	Kabara	34.94	32.57	100	19	25	13	12	9	540	50	60	70	75	53	138	1	27	21
33	Givat-Koach	34.92	32.03	75	20	26	12	14	12	540	50	50	64	65	105	160	1	32	26
34	W.Siverek	39.25	37.70	620	17	31	4	27	-	449	62	-	33	-	-	-	5	-	-
35	E.Siverk	39.44	37.91	950	12	25	1	24	-	588	68	-	51	-	-	-	5	-	-
37	N. Diyarbakir	40.06	38.13	720	15	28	3	25	-	516	75	-	42	-	-	-	5	-	-

Symbols of Variables-- Geographical: *Ln* Longitude, *Lt* latitude, *Al* altitude;

Temperature: *Tm* mean annual temperature, *Ta* mean August temperature, *Tj* mean January temperature, *Td* seasonal temperature difference, *Tdd* day-night temperature difference, *Trd* mean number of tropical days;

Water availability: *Rn* mean annual rainfall, *Rd* mean number of rainy days, *Hu-an* mean annual humidity, *Hu-14* mean humidity at 14:00 h, *Dw* mean number of dew nights in summer, *Ev* mean annual evaporation, *Rv* mean inter-annual variability of rainfall, *Rr* mean relative variability of rainfall;

Edaphic: *So* soil type, 1 = terra-rossa (t.r.); 2 = rendzina; 5 = basalt.

### Genomic DNA extraction and SNP genotyping

Young leaves from each accession were collected and frozen in liquid nitrogen. Genomic DNA was isolated using a modified SDS method according to Peng et al. [32]. The extraction buffer (pH 7.8–8.0) consisted of 500 mM sodium chloride (NaCl), 100 mM tris (hydroxymethyl) aminomethane hydrochloride (Tris–HCl) pH 8.0, 50 mM ethylene diamine tetraacetic acid (EDTA) pH 8.0, 0.84% (w/v) SDS, and 0.38% (w/v) sodium bisulfate.

The 200 wild emmer wheat accessions were genotyped with 1,536 SNP markers. These SNPs discovered in a panel of 32 lines of tetraploid and hexaploid wheat were downloaded from the Wheat SNP Database (http://wheat.pw.usda.gov/SNP/new/index.shtml). A detailed procedure of SNP selection and assay design have been described by Akhunov et al. [27,28] and Chao et al. [33]. Briefly, a total of 150 ng of genomic DNA per genotype was used for Illumina SNP genotyping at the Genome Center of University of California, Davis (http://www.genomecenter.ucdavis.edu/dna_technologies) using the Illumina Bead Array Platform and Golden Gate Assay following the manufacturer’s protocol [34]. The fluorescence images of an array matrix carrying Cy3- and Cy5- labeled beads were generated with the two-channel scanner. The ratio of the intensity of Cy3 and Cy5 fluorescence is used to determine the allelic state at an SNP site. Golden Gate genotyping reaction performed on polyploid wheat genomic DNA is expected to produce Cy3/Cy5 fluorescence ratios that differ from those expected for a diploid. Due to the bottleneck in the formation of tetraploid wheat, there was virtually no polymorphism introduced from the A or B genome ancestor. Thus all mutations arose after the formation of the found tetraploid population. The rate of spontaneous mutation is extremely low, 10^-8^–10^-9^ mutations/site/year in eukaryotic genomes. Therefore, two-mutation event occurred simultaneously in both the A and B genomes at a given nucleotide site is negligible. Considering the nature of self-pollination in emmer wheat, there will be only two genotypes for the accessions involved, for example, A— > T mutation in the A-genome yields a derived T base and an A/T SNP. In the B-genome, the ancestral A base remains unchanged. Hence, the SNP results in two homozygous genotypes, AAAA and TTAA. The ratio of A:T bases in these two genotypes are 1:0 and 1:1.

Subsequent genotype calling was carried out using Illumina’s BeadStudio software v.3. The accuracy of the genotype call was manually evaluated for the misclassification of homozygous and heterozygous clusters using the software’s clustering algorithm. This step proved critical for reducing the genotyping error rate associated with peculiarities of clustering patterns in polyploidy wheat [27,33].

### Genetic diversity and genetic structure

POWERMARKER Ver. 3.25 was used to evaluate genetic diversity [35]. The genetic parameters included Nei’s gene diversity and polymorphism information content (PIC). Nei’s gene diversity was defined as the probability that two randomly chosen alleles from the population are different [36]. PIC values provide an estimate of the probability of finding polymorphism between two random samples in the germplasm.

In order to have a better insight into the genetic structure of wild emmer wheat, we applied the Bayesian model-based clustering algorithm implemented in STRUCTURE 2.2 [37]. Admixture and correlated allele frequency models were employed with the number of clusters (*K*) ranging from 1 to 12. For each *K*, five runs were carried out. Burn-in time and replication number were both set to 100,000 for each run. The optimal value of *K* was determined using the Δ*K* method [38] and by inspecting the relationship between the log probability of the data and *K*.

The correlation between shared-allele distance and geographic distance (measured in kilometers) among populations was performed using the Mantel test, implemented in the GENEALEX6.0 software [39].

### Population differentiation and detection of outliers

Population differentiation and significance were assessed by calculating pairwise *F*_*ST*_ values for all population pairs using Arlequin 3.5 software [40]. Analysis of molecular variance (AMOVA) was performed to estimate the variance between populations and among accessions within populations, also implemented in the Arlequin 3.5 software. Significance levels for variance components and *F*_*ST*_ statistics were estimated using 16,000 permutations.

We also used Arlequin 3.5 to detect outlier loci taking into account the hierarchical structure of the populations, in which populations are divided into groups according to their genetic structure revealed by STRUCTURE analysis. The analysis was performed with 20,000 simulations under a hierarchical island model with 10 groups of 100 demes. The joint null distribution of *F*_*ST*_ and heterozygosity (heterozygosity within populations divided by (1- *F*_*ST*_)) was obtained according to Excoffier and Lischer [40]. Based on *F*_*ST*_ values that fall outside of the 99% confidence interval, candidate loci under positive selection were used for further analysis.

### Statistical tests

SPSS V.13.0 program (http://www.spss.com) was used to perform statistical analyses. The significance of differences for Nei’s gene diversity and PIC among chromosomes was tested by estimating a 95% confidence interval (CI) of the genome mean, which was calculated using bootstrap analysis with 1,000 replications. Chromosome means outside of the 95% CI were declared significantly different from the genome mean [28].

Multiple regression analysis was performed to investigate the relationship between environmental variables and SNP allele frequencies, and detect the best predictors of gene diversity and PIC index [14,15]. Nei’s gene diversity, PIC, and SNP allele frequencies were employed as dependent variables in the model, respectively; and geographic, climatic and edaphic factors served as independent variables. The following ecogeographical factors were included in the analysis. *Geographical* [longitude (Ln), latitude (Lt), and altitude (Al)], *climatic* [*temperature —* annual (Tm), January (Tj), August (Ta), seasonal temperature difference (Td), daily temperature difference (Tdd); number of tropical days (Trd), evaporation (Ev); *moisture —* annual rainfall (Rn), number of rainy days (Rd), number of dewy nights in summer (Dw), annual humidity (Huan), humidity at 14:00 (Hu14), inter-annual rainfall variation (Rv), coefficient of variation in rainfall (Rr)], and *edaphic* dummy variables [one per each of the soil types: basalt (Ba), rendzina (Ren) and terra rossa (Tr)]. The analysis was conducted using 21 of the examined wild wheat populations. Populations from Turkey including W. Siverek, E. Siverek, and N. Diyarbakir with many missing data and Mt. Hermon, a cold desert with the highest rainfall were excluded from this analysis in order to minimize the errors or the bias caused by extreme climate conditions.

## Results

### SNP marker quality and genomic distribution

Genotyping of 200 wild emmer wheat accessions with multiplexed 1,536 Illumina Golden Gate SNP assay generated 307,200 genotypic data points. Out of the 1,536 SNPs presented in our oligonucleotide pool assay (OPA), 1,371 (89.3%) SNPs with high quality genotype calls were obtained, while the other 10% failing to generate clear genotype clustering were removed. Out of the 1,371 scoreable SNP markers, 266 were monomorphic across all the 200 accessions and the overall polymorphism rate was 80.6%. Marker distribution, Nei’s gene diversity, and PIC values calculated for each chromosome and genome were presented in Table 2.

**Table 2:** Genomic distribution and diversity index of 1,105 polymorphic SNP markers in a set of 200 wild emmer wheat accessions from Israel and Turkey

**Chromosome**	**No. of SNP markers**	**No. of polymorphic markers**	**Gene diversity**	**PIC**
A genome				
1A	114	98 (85.96%)	0.1498*	0.1271*
2A	98	84 (85.71%)	0.1823	0.1507
3A	98	78 (79.59%)	0.2024*	0.1651*
4A	124	102 (82.26%)	0.1721	0.1440
5A	85	66 (77.65%)	0.1555*	0.1304*
6A	125	90 (72.00%)	0.1909*	0.1562*
7A	135	101 (74.81%)	0.1595	0.1315*
Subtotal/Mean	769	613 (79.71%)	0.1733	0.1443
B genome				
1B	100	88 (88.00%)	0.2140	0.1768
2B	88	71 (80.68%)	0.1995	0.1661
3B	67	55 (82.09%)	0.1879	0.1589
4B	75	60 (80.00%)	0.1733*	0.1466*
5B	76	57 (75.00%)	0.1498*	0.1273*
6B	105	90 (85.71%)	0.2253*	0.1880*
7B	102	79 (77.45%)	0.1897	0.1560
Subtotal/Mean	602	492 (81.73%)	0.1975	0.1649
Hemoeologous				
1	214	186 (86.92%)	0.1797	0.1505
2	186	155 (83.33%)	0.1915	0.1572
3	165	133 (80.61%)	0.1966*	0.1622*
4	199	162 (81.41%)	0.1720*	0.1454
5	161	123 (76.40%)	0.1531*	0.1292*
6	230	180 (78.26%)	0.2079*	0.1731*
7	237	180 (75.95%)	0.1724	0.1432*
Total/Grand mean	1371	1105 (80.60%)	0.1841	0.1530

* Means that is outside of the 95% bootstrap confidence interval of the genome mean.

Polymorphic SNP loci were not evenly distributed across the seven homoeologous groups, and coverage, number of marker loci per group, ranged from 123 in group 5 to 186 loci in group 1. Differences between homoeologous groups were significant (*P* < 0.05) for gene diversity and PIC (Table 2). Nei’s gene diversity varied from 0.1531 in group 5 to 0.2079 in group 6 with an average of 0.1841. The PIC value ranged from 0.1292 in group 5 to 0.1731 in group 6 with an average of 0.1530.

Of the polymorphic loci, 613 and 492 were located in A and B genomes of wild emmer wheat, respectively. As shown in Table 2, the higher genetic diversity was detected in genome B with Nei’s gene diversity and PIC values of 0.1975 and 0.1649, respectively, while 0.1733 and 0.1443 for genomes A, respectively. This difference between genome A and B was not statistically significant for both gene diversity (*t* = 1.762, *P* = 0.129, paired *t* test) and PIC (*t* = 2.126, *P* = 0.078, paired *t* test). In the genome A, chromosome 3A and 6A had higher genetic diversity and chromosome 1A and 5A had lower genetic diversity than the genome-wide average in the analyzed germplasm (Table 2). In the genome B, genetic diversity was lower in chromosome 4B and 5B than the genome-wide average, while genetic diversity was higher in chromosome 6B than the genome-wide average (Table 2).

### Genetic diversity

Proportion of polymorphic loci, gene diversity, and PIC of the 25 wild emmer wheat populations were summarized in Table 3. Among 25 populations, genetic diversity estimates exhibited remarkable variations, with Nei’s gene diversity ranging from 0.1101 (Qazrin) to 0.2583 (Daliyya) and PIC ranging from 0.0899 (Qazrin) to 0.2221 (Daliyya), respectively. Similarly, genetic diversity pattern was also reflected by the percentage of polymorphic loci within a population. The population of Daliyya had the highest percentage of polymorphic loci (*P* = 81.45%), followed by N. Diyarbakir (55.75%) and Yehudiyya (51.49%), whereas the polymorphic loci of Rosh-Pinna and Qazrin were the least (31.49-32.49%).

**Table 3:** Summary of genetic parameters revealed by 1,105 polymorphic SNP markers in 25 populations from Israel and Turkey

**Population code**	**Place of origin**	**Sample size**	**Polymorphism rate**	**Gene diversity**	**PIC**
1	Hermon	4	47.24%	0.1905	0.1515
5	Qazrin	10	32.49%	0.1101	0.0899
7	Yehudiyya	10	51.49%	0.1274	0.1082
8	Gamla	10	41.09%	0.1515	0.1212
9	Rosh-Pinna	4	31.49%	0.1149	0.0923
10	Ammiad	9	57.56%	0.1761	0.1466
11	Tabigha	9	40.90%	0.1489	0.1197
16	Mt. Gilboa)	6	37.83%	0.1536	0.1229
17	Mt. Gerizim	8	41.81%	0.1546	0.1249
18	Gitit	9	46.33%	0.1634	0.1319
19	Kokhav-Hashahar	7	46.43%	0.1627	0.1308
20	Taiyiba	8	41.45%	0.1445	0.1165
21	Sanhedriyya	10	42.53%	0.1497	0.1214
22	Bet-Meir	8	33.94%	0.1237	0.0987
23	J’aba	9	47.06%	0.1588	0.1292
24	Amirim	5	34.75%	0.1354	0.1081
27	Nesher	8	46.43%	0.1547	0.1258
28	Beit-Oren	7	37.92%	0.1403	0.1127
29	Daliyya	8	81.45%	0.2583	0.2221
30	Bat-Shelomo	9	49.32%	0.1644	0.1338
31	Kabara	6	43.44%	0.1563	0.1259
33	Givat-Koach	9	44.62%	0.1511	0.1225
34	W. Siverek	10	47.87%	0.1629	0.1322
35	E. Siverk	7	45.61%	0.1613	0.1306
37	N. Diyarbakir	10	55.75%	0.1924	0.1569
	Total/Grand mean	200		0.1842	0.1532

### Genetic relationships

**Figure 2:**
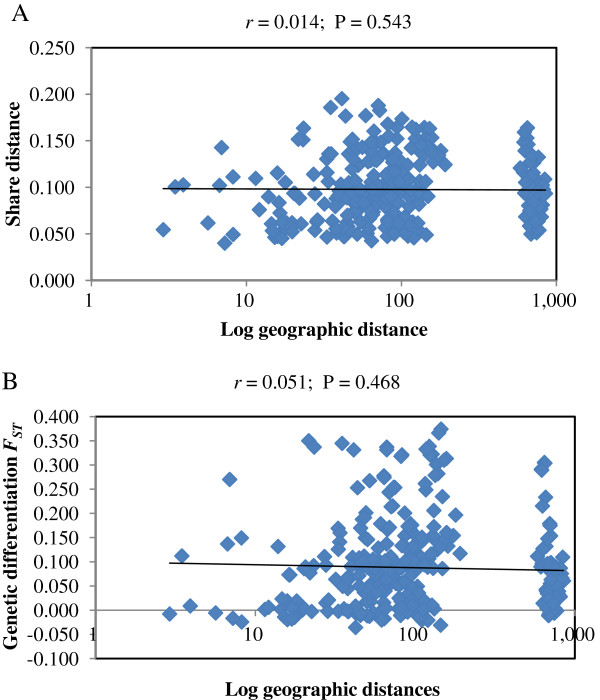
**Mantel test showing the relationship of genetic and geographic distances. (A)** Relationship between shared-allele distance and geographic distances, and **(B)** relationship between population differentiation *F*_*ST*_ and geographic distances.

Genetic distances (D) were calculated for all the population pairs, based on the shared-allele distance (Additional file 1: Table S1). The highest genetic distance (0.1953) was obtained between populations of Hermon and Yehudiyya, whereas the most related populations were Qazrin and Yehudiyya with a genetic distance of 0.0401. However, lower D values (D < 0.050) were observed between some populations from different areas, and, for the most part, the estimates of D value were geographically independent, as revealed by Mantel test (*r* = 0.014, *P* = 0.543; Figure 2A). These results suggest that geographic distance alone may not explain inter-population genetic divergence.

### Genetic structure

**Figure 3:**
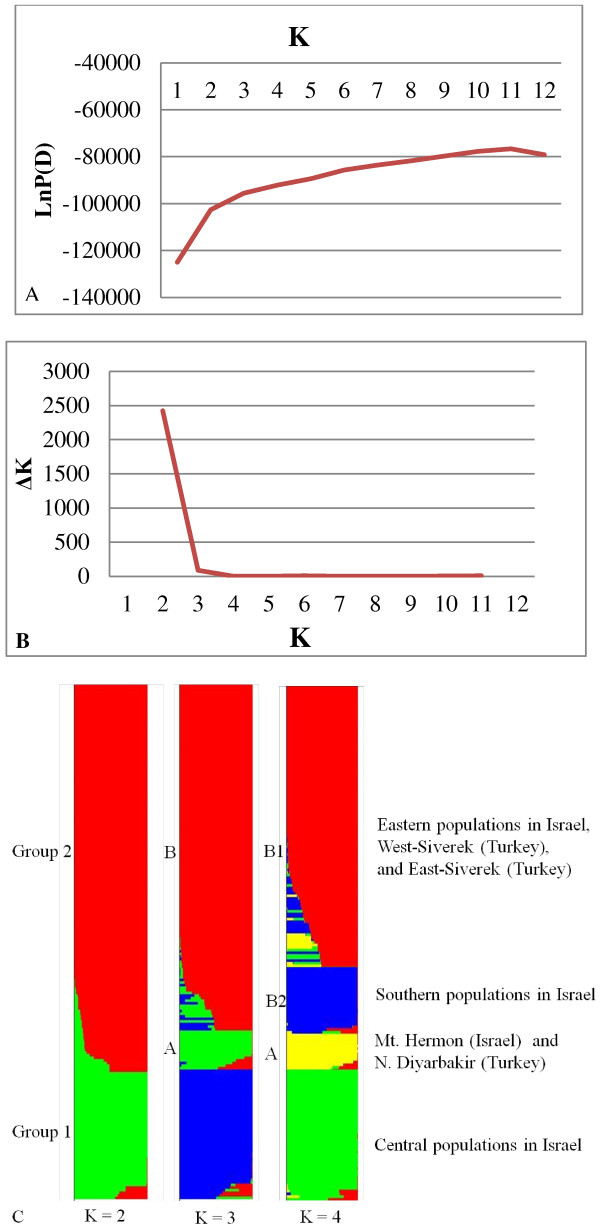
**Genetic structure of 25 wild emmer wheat populations from Israel and Turkey. (A)** Evolution of the natural logarithm probability of the data against *K*; **(B)** Magnitude of Δ*K* for each *K* value; **(C)** Clustering for *K* = 2 to 4 for the entire set of wild emmer wheat.

SNP genotyping data were used for genetic structure analysis, using the Bayesian clustering model implemented in the STRUCTURE software. The estimated log probability (LnP(D)) increased continuously with increasing *K*, and there was no critical *K* value that clearly defines the number of populations (Figure 3A). We applied the rate of change in the Napierian logarithm probability relative to standard deviation (Δ*K*). The results suggested that the optimal value of *K* was 2 (Figure 3B). When *K* = 2, the largest number of accessions (188/200 = 94%) assigned to a specific cluster with a probability higher than 80% was obtained, and only 6% were classified as admixed. However, percentage of unassigned genotypes, classified as admixed, increased continuously with *K,* and this percentage is 8.5%, 14%, and 42% when *K* = 3, 4 and 5, respectively. Hence, the clustering diagrams with *K* ranging from 2 to 4 are presented in Figure 3C.

When *K* = 2, the analyzed wild emmer wheat populations can be divided into two genetically distinct groups (Group I and Group II) (Figure 3C). Group I was composed of all the central populations from Israel including Tabigha, Ammiad, Rosh-Pinna, Qazrin, and Yehudiyya. Group II consisted of all the marginal populations from Israel (west marginal populations: Amirim, Nesher, Beit-Oren, Daliyya, Bat-Shelomo, Kabara, and Givat-Koach; south-east marginal population: Gitit, Mt. Gerizim, Mt. Gilboa, Kokhav-Hashahar, Taiyiba, Bet-Meir, Sanbedriyya, and Jaba; and north marginal population: Hermon) and Turkey populations (W. Siverek, E. Siverk, N. Diyarbakir) (Figure 3C). When *K* = 3, Group I was the same as in the previous analysis, but Group II was subdivided into two subgroups (Group A and Group B) (Figure 3C). That is, accessions from Hermon and N. Diyarbakir were separated from Group II. When *K* = 4, only Group B was further subdivided into two subgroups (Group B1 and Group B2), and accessions from south marginal populations including Taiyiba, Bet-Meir, Sanbedriyya and Jaba were clustered together (Figure 3C).

### Genetic differentiation of populations

Population differentiation was assessed with an analysis of molecular variance (AMOVA). The AMOVA revealed that individuals within populations are highly genetically differentiated in relation to individuals among populations, which is reflected by a higher proportion of variance within populations than among populations. Ninety percent of the genetic variation resided among accessions within populations, while a small (9.82%) but significant (*P* < 10^-5^) portion of the variation resided between populations (Table 4). Moreover, fixation index (*F*_*ST*_ = 0.098) was highly significant (*P* < 10^-5^) as indicated by permutation test. These results indicate that differentiation between populations has truly occurred.

**Table 4:** Analysis of molecular variance (AMOVA) in a set of 200 wild emmer wheat accessions representing 25 populations from Israel and Turkey

**Source of variation**	**d.f.**	**Sum of squares**	**Percentage of variation (%)**	***P*-value**
Among Populations	24	6068.53	9.82	<10^-5^
Within populations	375	34620.12	90.18	<10^-5^
Total	399	40688.66	100	
Fixation index, *F*_*ST*_ = 0.098				<10^-5^

Note: *P*-value was obtained through significance test using 16,000 permutations.

Indeed, coefficients of population differentiation (*F*_*ST*_) were also calculated for pairwise comparisons of the 25 populations (Additional file 2: Figure S1). The *F*_*ST*_ values for all 300 pairs ranged from -0.0356 to 0.3502, with 126 pairs showing significant genetic differentiation (*P* < 0.05). Forty-four out of 126 pairs showed strong genetic differentiation (*F*_*ST*_ > 0.2). However, genetic differentiation between populations was independent of geographical distances between the sites of collection, as revealed by the Mantel test (*r* = 0.051, *P* = 0.468; Figure 2B). This finding suggests that there is no evidence for an isolation-by-distance pattern of population differentiation in wild emmer wheat.

**Figure 4:**
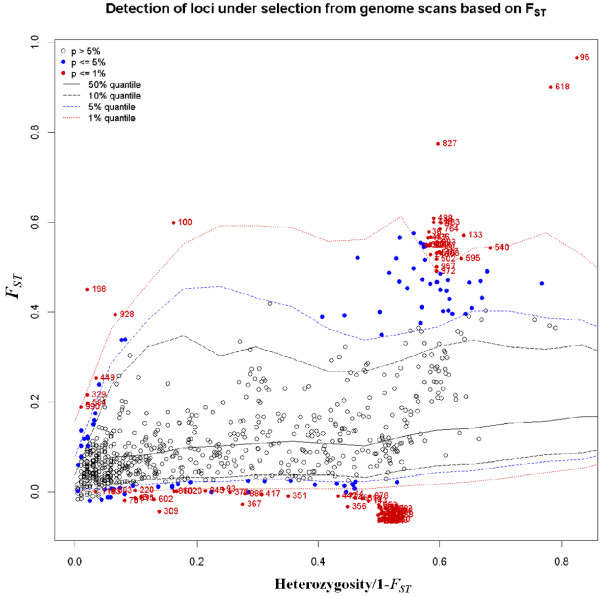
**Detection of outlier SNPs under the hierarchical structure model using Arlequin 3.5.***F*_*ST*_: locus-specific genetic divergence among populations; Heterozygosity/1- *F*_*ST*_: a modified measure of heterozygosity per locus. Loci significant at the 1% level are indicated by red dots.

**Figure 5:**
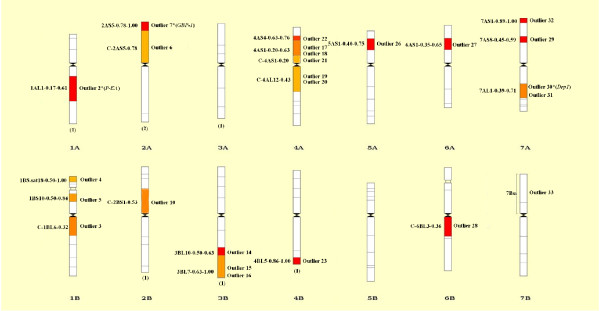
**Chromosomal distribution of 33 outlier loci subjected to positive selection.** The codes of mapped loci are shown on the right of each chromosome and the intervals are indicated on the left. Details of codes are presented in Table 5. Candidate loci from known genes of wheat were indicated by *, and these known genes subjected to positive selection were listed after each loci. The number in parentheses at the bottom of each chromosome is the number of EST loci mapped in that chromosome without knowing the exact bin. Only these bins with mapped loci are indicated.

Adaptive differentiation has conventionally been identified from differences in allele frequencies among different populations, reflected by *F*_*ST*_*,* an appropriate genetic parameter for measuring population differentiation and hence identifying outlier loci. In this study, outlier loci were identified using the *F*_*ST*_-based method that considers the hierarchical structure in order to minimize the number of false-positive loci. We focused on the results when *K* = 2, since the model-based approach of STRUCTURE indicated that *K* = 2 was assumed to be optimal. A total of 102 outlier loci were identified when *K* = 2. Among these, 69 loci were candidates for balanced selection, while only 33 loci were candidates for being subjected to positive selection (Figure 4). Chromosomal distributions of these loci were shown in wheat chromosome bin maps (Figure 5). A high portion of these loci (54.5%) were located in chromosomes 1B, 2A, 3B, 4A, and 7A (Table 5; Figure 5).

**Table 5:** ESTs and the plausible functions in the homologous ESTs outlier loci among populations

**SNP marker and EST**			**Gene function and homologus EST**			
**Code**	**SNP markers**	**Map position**	**Function**	**Accession no.**	**Identity**	**E-value**
Outlier 1	BE605063_1_A_Y_252	1A	Uncharacterized LOC100821025, *B. distachyon*	XM_003574117.1	89%	0
Outlier 2*	BE494527_1_A_Y_449	1AL1-0.17-0.61	Phosphoethanolamine methyltransferase, *T. aestivum*	AY065971.1	96%	3e-86
Outlier 3	BG314205_1_B_33	C-1BL6-0.32	Uncharacterized LOC100839226, *B. distachyon*	XM_003574093.1	86%	0
Outlier 4	BE637971_1_B_Y_181	1BS.sat18-0.50-1.00	Uncharacterized LOC100831964, *B. distachyon*	XM_003573654.1	85%	2e -108
Outlier 5	BE443797_1_B_436	1BS10-0.50-0.84	26S proteasome non-ATPase regulatory subunit 13-like, *B. distachyon*	XM_003574348.1	89%	0
Outlier 6	BE445242_2_A_362	C-2AS5-0.78	Uncharacterized LOC100842418, *B. distachyon*	XM_003562526.1	90%	0
Outlier 7*	BE518440_2_A_Y_187	2AS5-0.78-1.00	GTP-binding protein (GBP-1), *T. aestivum*	DQ489316.1	97%	0
Outlier 8	BE406351_2_A_Y_124	2A	Probable glycerophosphoryl diester phosphodiesterase 2, *B. distachyon*	XM_003579903.1	91%	4e-139
Outlier 9	BE406351_2_A_76	2A	Probable glycerophosphoryl diester phosphodiesterase 2, *B. distachyon*	XM_003579903.1	91%	4e-139
Outlier 10	BE404601_2_B_Y_66	C-2BS1-0.53	Uncharacterized LOC100826320, *B. distachyon*	XM_003562643.1	85%	4e1-15
Outlier 11	BE499362_2_B_Y_257	2B	Probable signal peptidase complex subunit 2-like, *B. distachyon*	XM_003579773.1	91%	0
Outlier 12	BG263769_3_A_Y_343	3A	Uncharacterized LOC100822853, *B. distachyon*	XM_003569549.1	92%	9e-151
Outlier 13	BE445508_3_B_Y_273	3B	Aegilopoides isolate D58c hypothetical protein, *T. monococcum*	HM539557.1	99%	0
Outlier 14	BE517914_3_B_340	3BL10-0.50-0.63	Ras-related protein RABE1a-like, *B. distachyon*	XM_003564523.1	94%	2e-178
Outlier 15	BE424246_3_B_131	3BL7-0.63-1.00	cDNA, clone: WT006_E06, *T. aestivum*	AK333352.1	99%	0
Outlier 16	BE517732_3_B_294	3BL7-0.63-1.00	KH domain-containing protein, *B. distachyon*	XM_003564748.1	82%	5e-141
Outlier 17	BE443973_4_A_Y_130	4AS1-0.20-0.63	Probable dolichyl pyrophosphate Man9GlcNAc2 alpha-1, 3-glucosyltransferase, *B. distachyon*	XM_003557947.1	90%	0
Outlier 18	BE443973_4_A_105	4AS1-0.20-0.63	Probable dolichyl pyrophosphate Man9GlcNAc2 alpha-1, 3-glucosyltransferase, *B. distachyon*	XM_003557947.1	90%	0
Outlier 19	BE490599_4_A_Y_132	C-4AL12-0.43	cDNA, clone: WT003_N17, cultivar: Chinese Spring, *T. aestivum*	AK332386.1	92%	2e-82
Outlier 20	BE497184_4_A_Y_109	C-4AL12-0.43	Putative methylsterol monooxygenase DDB_G0269788-like, *B. distachyon*	XM_003577145.1	91%	0
Outlier 21	BE591861_4_A_Y_848	C-4AS1-0.20	KH domain-containing protein At4g18375-like, *B. distachyon*	XM_003557826.1	90%	5e-174
Outlier 22	BE404717_4_A_Y_280	4AS4-0.63-0.76	Kelch motif family protein, *Z. mays*	NM_001155181.1	79%	5e-50
Outlier 23	BE446161_4_B_Y_162	4BL5-0.86-1.00	cDNA, clone: SET2_P19, *T. aestivum*	AK335999.1	99%	0
Outlier 24	BF482356_4_B_Y_504	4B	Ubiquitin carboxyl-terminal hydrolase 12-like, *B. distachyon*	XM_003577385.1	91%	3e-171
Outlier 25	BF482216_4_B_Y_57	4BL5-0.86-1.00	Endoribonuclease Dicer homolog 1-like, *B. distachyon*	XM_003558898.1	91%	0
Outlier 26	BE443538_5_A_1436	5AS1-0.40-0.75	Pollen-specific protein SF3-like, *B. distachyon*	XM_003575978.1	93%	0
Outlier 27	BG275060_6_A_Y_309	6AS1-0.35-0.65	Peroxisomal fatty acid beta-oxidation multifunctional protein, *B. distachyon*	XM_003572368.1	92%	0
Outlier 28	BE590521_6_B_N_331	C-6BL3-0.36	Adenine phosphoribosyltransferase 2-like, *B. distachyon*	XM_003575301.1	90%	4e-75
Outlier 29	BE498662_7_A_Y_513	7AS8-0.45-0.59	Pantothenate kinase 4, *Z. mays*	NM_001156082.1	87%	3e-137
Outlier 30*	BE488670_7_A_Y_73	7AL1-0.39-0.71	Spermidine synthase, *T. aestivum*	HQ121400.1	99%	0
Outlier 31	BF482529_7_A_304	7AL1-0.39-0.71	50S ribosomal protein L1-like, *B. distachyon*	XM_003563790.1	86%	8e-143
Outlier 32	BF292264_7_A_712	7AS1-0.89-1.00	GDT1-like protein, *B. distachyon*	XM_003574474.1	94%	4e-52
Outlier 33	BE518436_7_B_Y_671	7Bs	BRCA1-A complex subunit BRE-like, *B. distachyon*	XM_003563526.1	88%	3e-176

Candidate loci from known genes of wheat were indicated by *.

The SNP markers used in the present study were derived from genomic sequences amplified from conserved primers, which were located in exons and were designed on the conserved sequences between wheat EST and rice genomic sequences [28,41]. A putative function of these 33 loci thus may be deduced based on comparison of the underlying genes to a protein sequence database. Among the 33 loci, P-EA (phosphoethanolamin emethyltransferase), GBP-1 (GTP-binding protein), and SPDS (Spermidine synthase) were found to be under positive selection (Table 5; Figure 5).

### Association between markers and ecogeographical factors

As shown in Additional file 3: Table S2, the water-availability factor alone explained a significant proportion of the diversity revealed by SNP markers. The best two variable predictors of gene diversity and PIC index, explaining significantly 0.29–0.30 of their variance (P < 0.01), were Rv and Ev (inter-annual rainfall variation and evaporation). A three-variable combination involving RvEvHu14 (inter-annual rainfall variation, evaporation, and humidity at 14:00), accounted significantly (p < 0.01) for 0.48-0.49 of the variance in gene diversity and PIC index.

Out of 1,105 polymorphic SNP markers, 755 including 33 outlier loci subjected to positive selection were significantly correlated with ecogeographical factors, single or in combination, for allele frequency (Additional file 3: Table S2). Environmental factors including geography, temperature, and water-availability factors, singly or in combination, explained a significant proportion of variation in SNP allele frequency, from 0.2 to 0.9. Based on correlation of allele frequency with environmental factors, the 755 SNP markers can be classified into several categories in terms of their chosen ecogeographical predictors (Additional file 3: Table S2): 

a) Water factors (Huan, Rr, Dw, Rd, Rv, Rn): 316;

b) Geographic factors (Lt, Al): 30;

c) Temperature factors (Td, Tm, Trd, Tj, Tdd, Ta, Ev): 69;

d) Geographic factors + water factors (Lt, Huan, Rv, Rr, Rn): 121;

e) Water and temperature factors (Rr, Rv, Rd, Hu14, Td, Tm, Trd, Tj, Tdd): 80;

f) Geographic factors + temperature factors (Lt, Td, Tdd, Tm): 70; and

g) Geographic factors + temperature + water (Lt, Td, Tdd, Huan, Rv, Rd, Dw): 69.

## Discussion

### Genetic diversity revealed by EST-related SNP markers

**Figure 6:**
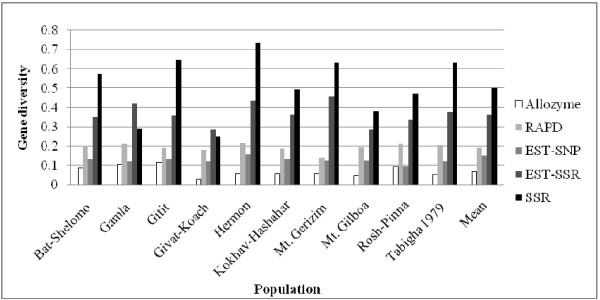
Gene diversity (Nei 1978) profiles of allozyme, RAPD, EST-SSR, SSR, and EST- related SNP loci in 10 wild emmer wheat populations from Israel and Turkey.

Average Nei’s gene diversity and PIC of the 25 populations of wild emmer wheat in this study were 0.1841 and 0.1530, respectively. Compared to those obtained previously with EST-SSR [42], SSR [15], RAPD [14], and allozyme [13], this level of genetic diversity is moderate. As shown in Figure 6, EST-related SNP markers were more polymorphic than allozyme loci, but lower than RAPD and SSR loci among the wild emmer wheat populations. Furthermore, a medium proportion of SNPs (31.49%-81.45%) were detected within populations indicating a moderate level of diversity within populations (Table 3). This result is expected, because of the more conserved nature of coding sequences sampled by EST-related SNP markers relative to non-coding sequences sampled by microsatellites and RAPDs. Another reason may be explained by the property of SNPs and the definition of gene diversity. SNP markers are mainly biallelic, the gene diversity and PIC thus cannot exceed 0.50, whereas the maximum can approach 1 for multi-allelic markers, such as SSRs. Despite these facts, our results show a sufficient level of variation when using EST-related SNP markers to carry out genetic structure and future association mapping analysis. Therefore, the result of this study provided evidence showing that the EST-related SNP markers may provide an opportunity to examine the functional diversity of germplasm collections, as reported by Chao et al. [29].

### Genetic structure of wild emmer wheat populations

This study presents the first genome-wide analysis on population structure of SNP genetic variation among natural populations in wild emmer wheat. Clustering based on Bayesian model showed that the grouping pattern is related to the ecogeographic distribution of the wild emmer wheat populations. All central populations collected from warm and humid environments in the Golan Plateau (Qazrin, Yehudiyya and Gamala) and near the Sea of Galilee (Tabigha, Ammiad and Rosh-Pinna) were separated from marginal populations when *K* = 2, 3 and 4, respectively (Figure 3C). Although marginal populations, collected across a wide geographic areas on the northern, eastern, and southern borders of wild emmer distribution, involving hot, cold and xeric peripheries, were clustered together when *K* = 2, while Mt. Hermon in Israel together with N. Diyarbakir in Turkey showed a clear separation from the other marginal populations when *K* = 3. This clustering may be explained by the similarity in ecological conditions. The two sites are located in mountains with relatively high altitude, 1300 m and 720 m, and similarly low winter temperature, 3°C and 3°C of mean January temperature, for Mt. Hermon and N. Diyarbakir, respectively (Table 1). Furthermore, Mt. Hermon is closer to N. Diyarbakir than the other Israeli populations (Figure 1). When *K* = 4, the south xeric populations, Taiyiba, Bet-Meir, Sanbedriyya and Jaba are clustered together, but clearly separated from the west mesic (Mediterranean) populations. These results suggest that ecological variables play an important role in shaping the genetic structure of wild emmer wheat.

Indeed, SNP-based genetic distances were found to be independent on the geographical distances, as revealed by the Mantel test (*r* = 0.014, *P* = 0.543; Figure 2A). For example, the two most geographically distant populations, J’aba and N. Diyarbakir (850 km apart), exhibited a low value of genetic distance (0.067), while two adjacent populations, Gamla and Yehudiyya (7 km apart), showed a relatively high value of genetic distance (0.137). This suggests that geographic distance alone may not explain inter-population genetic divergence, which rules out an isolation-by-distance model. Hence, genetic distances of some populations may have a closer association with ecological variables relative to geographical distribution.

### Genetic differentiation of populations

Natural habitats of wild emmer wheat differ from one another in a large number of variables such as macro- and micro-climate, topography, soil type, etc. Such local ecogeographic differentiation may enhance plant populations to evolve local ecological adaptations that provide an advantage under the prevailing conditions [2,16]. Adaptive differentiation has conventionally been identified from differences in allele frequencies among different populations, summarized by an estimate of *F*_*ST*_[43,44]. This *F*_*ST*_ approach has been applied to many crops, such as the common bean [45] and tomato [46], and markers identified by using a *F*_*ST*_-outlier method in these species tended to map to genome regions with known genes and quantitative trait loci related to domestication.

In the present study, we identified 33 candidate loci under positive selection based on *F*_*ST*_ values that displayed differentiation higher than the 99% limit of the confidence interval (Figure 4). These loci may be directly under selection, but more likely mark regions of the genome that have been selected during evolution, because some candidate loci clustered in the same chromosomal regions, such as outlier 17 and 18, and outlier 30 and 31 (Table 5; Figure 5). The loci we identified have a disproportional bias with 54.5% mapping to chromosomes 1B, 2A, 3B, 4A and 7A (Table 5; Figure 5). This observation suggests that there are ‘hot spots’ for directional selection in genome of wild emmer wheat. An analysis of wheat’s chromosome maps by Map Viewer (http://www.ncbi.nlm.nih.gov/projects/mapview/) indicated that a large number of multiple fungal disease-resistance genes exist in chromosomes 1B, 2A, 3B, 4A and 7A, such as *Lr17*, *Lr20*, *Lr27*, *Lr28*, *Lr30*, *Lr38*, *Sr2*, *Sr7*, *Sr15*, *Sr21*, *Sr22*, *Sr38*, *Yr17*, *Pm1*, *Pm4*, and *Hd.* In addition, three genes including *P-EA*[47], *GBP-1*[48] and *SPDS*[49,50], which play important roles in plant responses to biotic and abiotic stresses or in plant growth and development in wheat [47-50], appear to be subjected to positive selection. This result suggested that the markers and genome locations we identified as outliers under positive selection were consistent with known patterns of selection that differentiated central populations from marginal populations. Large number of accessions from central populations located near the Sea of Galilee and the Golan Heights were resistant to stripe rust and powdery mildew, while marginal populations were collected across wide geographic areas on the northern, eastern and southern borders of wild emmer distribution, involving in hot, cold and xeric stress [1]. Such an objective assessment may provide a scalable means for comprehensive assessments of genetic variation within wild emmer wheat as emerging sequence data and improved genotyping platforms lead to larger datasets [46].

### Ecogeographical factors *vs.* population divergence and genetic structure

The organization and evolution of genetic diversity in nature at global, regional, and local scales are nonrandom and heavily structured; and are positively correlated with, and partly predictable by, abiotic and biotic environmental heterogeneity and stress [51], as shown earlier by allozyme and DNA markers. However, Prunier et al. recently found origin and evolution of adaptive polymorphisms in black spruce can be modified by historical events, hence affecting the outcome of recent selection and leading to different adaptive routes between intraspecific lineages [43]. In this study, we found that ecogeographical factors play an important role in shaping genetic structure and enhancing population divergence in wild emmer wheat from Israel and Turkey. Significant correlations between marker loci and ecogeographical factors were observed in the analyzed germplasm. Latitude, temperature, and water-availability factors, singly or in combination, explained a significant proportion in variation of SNP allele frequency (Additional file 3: Table S2). These findings suggest that natural selection could create regional divergence in wild emmer wheat. Especially, water-availability factors alone explained a significant proportion of genetic diversity revealed by SNP markers (Additional file 3: Table S2). The association of these factors with SNP-based genetic diversity was similar to that between allozyme variation and ecogeographical factors [13] and to that of latitude/altitude with RAPD and microsatellite diversity [14,15]. These results suggested that the operation of natural selection and the adaptive nature of genetic variation could be explained by the variation of ecological factors. The sharp regional gradient of climatic conditions from north to south in Israel, with increasing temperatures and decreasing water availability towards the semiarid zones in southern Israel play a major role as do microecological climatic and edaphic stresses [16,52]. That is also why latitude was found to be associated with frequency variation for most SNP allele (Additional file 3: Table S2). Therefore, natural selection appears to play a major role in generating adaptive structures coupling with environmental stresses in wild emmer wheat as in other organisms [53].

## Conclusions

The present work, using genome scan approach, presented strong evidence for adaptive genetic divergence in wild emmer wheat associated with ecological factors. Ecological factors, singly or in combination, explained a significant proportion in variation of SNP allele frequency. The SNPs could be classified into several categories of ecogeographical predictors. We identified a total of 33 loci under positive selection by using an *F*_*ST*_-outlier method. The markers and genome segments we identified as outliers under positive selection were consistent with known patterns of selection. These results suggested that ecological factors plaid an important evolutionary role in generating adaptive structures in wild emmer wheat. SNP markers are appropriate for detecting selectively-channeled adaptive genetic diversity in natural populations of wild emmer wheat. However, it will be greatly helpful to conduct functional studies to confirm the role of these outlier loci or genome segments in wild emmer wheat.

## Availability of supporting data

All the supporting data are included as additional files.

## Competing interests

The authors declare that they do not have any financial or non-financial competing interests exist.

## Authors’ contributions

JR carried out DNA extraction and data analysis, and drafted the manuscript. LC and DS carried out DNA extraction. FMY designed the SNP markers and worked on finalizing the manuscript. JW performed the SNP genotyping. YP and DFS carried out the field experiments. EN and AB worked on the manuscript; MCL was in charge of SNP design and genotyping work and participated in drafting the manuscript. JP was in charge of the entire research including experimental design, germplasm collection, outlining and finalizing the manuscript. All the authors read and approved the final version of the manuscript.

## Supplementary Material

Additional file 1: Table S1 Shared-allele distance between populations of wild emmer wheat. Click here for file

Additional file 2: Figure S1 Differentiation coefficients shown as pair-wise *Fst* among populations of wild emmer wheat. Click here for file

Additional file 3: Table S2 Coefficient of multiple regressions (R^2^) of genetic indices (genetic diversity indices and selected allele frequencies as the dependent variables) and environmental variables (as independent variables) in 25 populations of wild emmer wheat, *T. dicoccoides,* in Israel and Turkey. Click here for file
