# Supplementary figures and images for: SNP-revealed genetic diversity in wild emmer wheat correlates with ecological factors
Source: BMC Evol Biol. 2013 Aug 13;13:169. doi: 10.1186/1471-2148-13-169 (PMC3751623; doi:10.1186/1471-2148-13-169)

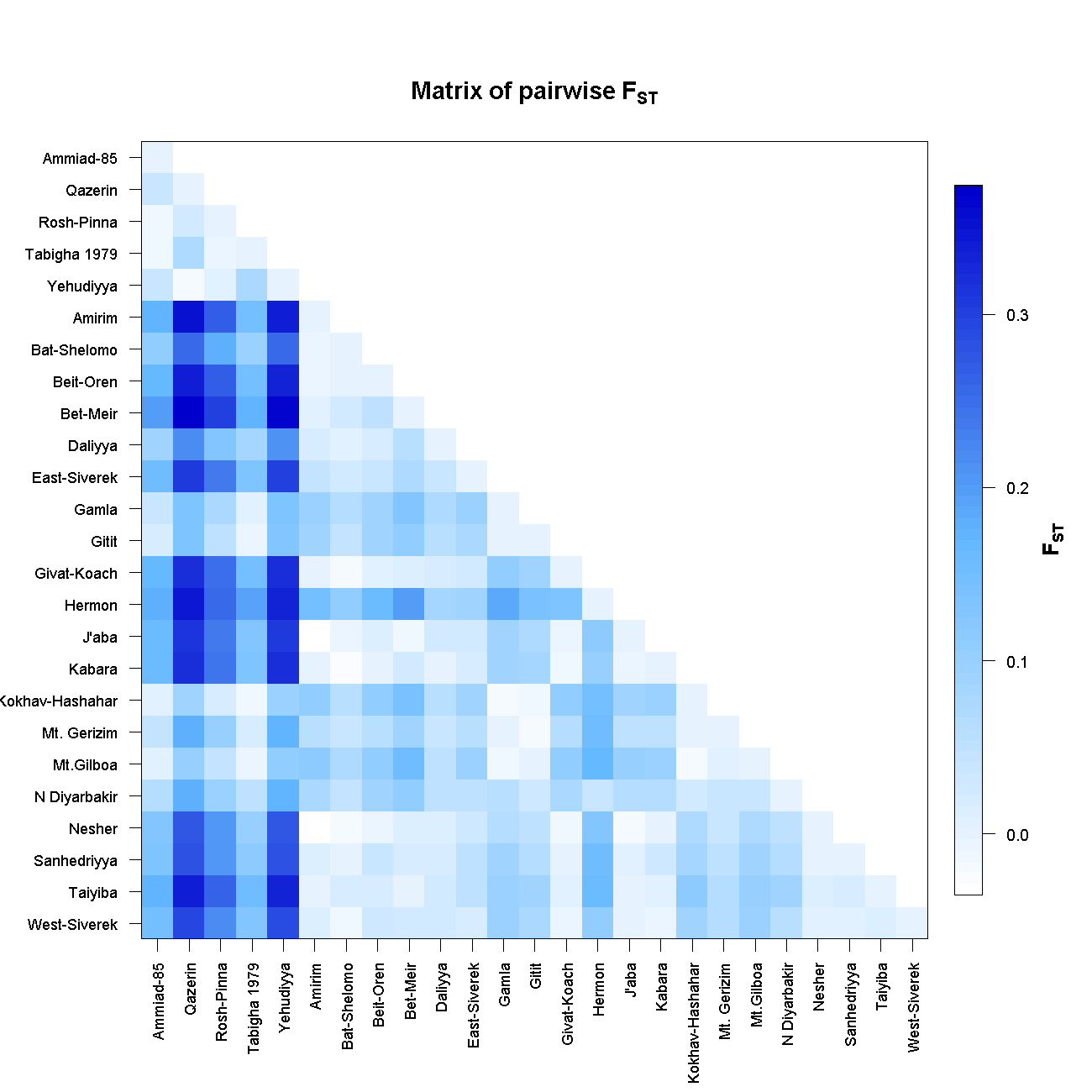

Supplement: Additional file 2: Figure S1 — Differentiation coefficients shown as pair-wise Fst among populations of wild emmer wheat. [file 1471-2148-13-169-S2.png]
